# Intensive Voice Treatment following Botulinum Neurotoxin Injection for a Speaker with Abductor Laryngeal Dystonia: An Exploratory Case Study

**DOI:** 10.1055/s-0044-1779509

**Published:** 2024-02-28

**Authors:** Mindy Schnell, Dianne Slavin

**Affiliations:** 1 Department of Communication Sciences and Disorders, Long Island University, Post, Greenvale, New York

**Keywords:** dysphonia, voice, LSVT, laryngeal dystonia, BoNT

## Abstract

Abductor laryngeal dystonia (ABLD) is a rare neurological voice disorder which results in sporadic opening of the vocal folds during speech. Etiology is unknown, and to date there is no identified effective behavioral treatment for it. It is hypothesized that LSVT LOUD®, which was developed to treat dysphonia secondary to Parkinson's disease, may have application to speakers with ABLD to improve outcomes beyond that with botulinum neurotoxin (BoNT) treatment alone. The participant received one injection of BoNT in each vocal fold 2 to 3 months prior to initiating intensive voice therapy via teletherapy. Objective measures of vocal loudness (dB sound pressure level), maximum phonation time, and high/low pitch frequency (Hz) were recorded in all treatment sessions and follow-up sessions. Over the course of treatment, the participant showed steady gains in phonation time, volume, pitch range, and vocal quality with a substantial reduction in aphonic voice breaks by the end of the treatment program. Perceptual symptoms of ABLD were nearly undetectable by the participant and the clinicians up to 12 months posttreatment, with no additional BoNT injections. The results suggest that LSVT LOUD® following BoNT was effective, with long-lasting improvement in vocal function, for this speaker with ABLD.

**Learning Outcomes** : As a result of this activity, the reader will be able to:

- Describe the characteristics of abductor laryngeal dystonia.
- Summarize the potential benefits of intensive vocal intervention in the treatment of abductor laryngeal dystonia.
- Discuss the potential value in applying LSVT LOUD to the treatment of abductor laryngeal dystonia.

Laryngeal dystonia (LD), formerly known as spasmodic dysphonia (SD) (Simonyan et al., 2021), is a rare neurological voice disorder/focal dystonia that affects the vocal folds. There are two types. The most common type, adductor laryngeal dystonia (ADLD), is an idiopathic focal dystonia that causes the vocal folds to intermittently close, often resulting in a strained-strangled quality and recurrent aphonia, primarily during voice onset (Cimino-Knight & Sapienza, 2001; Isetti, Xuereb, & Eadie, 2014). The abductor type (abductor laryngeal dystonia [ABLD]), also a focal dystonia, causes the vocal folds to sporadically open resulting in recurrent aphonia, breathy vocal quality, and reduced intelligibility (Braden & Hapner, 2008; Ludlow, 2011). Blitzer, Brin, and Stewart (1998) wrote that ABLD is characterized by spasms of the posterior cricoarytenoid (PCA) muscles, producing a breathy, effortful, hypophonic voice with abrupt termination of voicing, causing aphonic or whispered segments of speech. Simonyan et al. (2021) added that ABLD is a task-specific dysphonia affecting speech but does not impact whispering, yawning, laughing, or crying.

Numerous treatments for LD have been studied over the past 30 years. Blitzer et al. (1998) conducted a 12-year study of 900 patients with either ADLD or ABLD comparing improved vocal function following injections of botulinum toxin. The authors reported that 787 patients with adductor type had an average of 90% return of voice function lasting an average of 15.1 weeks. A total of 154 patients with abductor type had an average benefit of 66.7% of normal function lasting an average of 10.5 weeks. Based on these results, botulinum toxin injection was considered to be the treatment of choice for LD and remains so today.

Additional research includes a wide variety of potential treatments for LD. One potential treatment involves electric stimulation trials (Friedman, Grybauskas, Toriumi, & Applebaum, 1989; Pitman, 2014). Other investigations include vibration devices embedded in an external collar (Mahnan, Konczak, & Faraji, 2019), laryngeal vibrotactile stimulation (Khosravani et al., 2019; Mahnan, 2021), and deep brain stimulation to block the spasm neurologically (Honey et al., 2021). Additional studies with botulinum neurotoxin injections (BoNT) (Simpson, et al., 2008) and noninvasive brain stimulation (Chen et al., 2020) have been conducted with a small number of subjects and have reported mixed results. Since many of these treatments are experimental, invasive, or require expensive equipment not readily available, these may not be optimal treatments for LD. In addition, there are no FDA-approved pharmaceuticals or surgical treatments for LD. It should also be noted that most of the aforementioned research has focused on subjects with adductor LD, leaving those diagnosed with abductor LD with even fewer treatment options.

For decades, the standard treatment of care for LD is with botulinum toxin injection (BoNT) which must be repeated periodically (Blitzer et al., 1998; Klap et al., 1991; Lin & Sadoughi, 2020; Srirompotong et al., 2006; Watts, Nye, & Whurr, 2006; Watts, Whurr, & Nye, 2004; Watts, Truong, & Nye, 2008). While it has been reported that speakers with ADLD improved to an average of 97% (Whurr, Nye, & Lorch, 1998), it has been found that speakers with ABLD improved to an average of 70% of normal voice (Blitzer et al., 1992). Woodson, Hochstetler, and Murry (2006) reported less effectiveness with BoNT for ABLD than for ADLD, concluding that although injection into the PCA muscle can suppress abductor spasm, breathiness may persist. The results of the previously cited studies have been largely accepted as a satisfactory result by otolaryngologists. The question, however, is whether this achieved outcome, with a repeated invasive intervention every 3 to 4 months, is satisfactory to patients. In fact, in a review of the safety and efficacy of BoNT in the treatment of movement disorders, it was determined that there is insufficient evidence to support a conclusion of effectiveness of BoNT in ABLD (Simpson et al., 2008).

Murry and Woodson (1995) found that ADLD was treated most effectively with BoNT injection combined with reducing hyperfunctional vocal behaviors treated with voice therapy. Thus, the question is whether BoNT may be enhanced if combined with strengthening vocal behaviors as identified within intensive voice therapy similarly for ABLD.

Lee Silverman Voice Therapy (LSVT LOUD®) (Alharbi et al., 2019; Bryans et al., 2021; Sapir et al., 2002) is an intensive voice therapy based on the principles of brain plasticity. With a focus on intensity, repetition, intention, and recalibration of perceived speech loudness, LSVT LOUD has been demonstrated to be efficacious in treating the degenerative speech characteristics of Parkinson's disease (PD). These characteristics include reduced loudness, reduced intelligibility, and dysphonia. Researchers have documented positive results for patients with PD in each of these areas (Baumgartner, Sapir, & Ramig, 2001; Levy et al., 2020; Sapir et al., 2002; Smith et al., 1995). The goals of intensive voice therapy focus on strengthening the degenerating muscles of the vocal tract via rigorous exercise and heightened awareness.

This approach has proved effective in improving vocal motor function with patients with other neurologically based dysphonias. In recent years, a number of researchers have administered LSVT LOUD to patients with dysarthrias secondary to multiple sclerosis (Baldanzi et al., 2022; Sapir et al., 2001), ataxia (Lowit, Egan, & Hadjivassiliou, 2020; Sapir et al., 2003), stroke (Mahler, Ramig, & Fox, 2009), cerebral palsy (Boliek, Halpern, Hernandez, Fox, & Ramig, 2014; Fox & Boliek, 2012; Langlois et al., 2020; Moya-Galé et al., 2022; Reed, Cummine, Fox, & Boliek, 2017), and non-progressive dysarthria (Wenke, Cornwell, & Theodoros, 2010). Promising results have been reported for improved vocal loudness and articulation. As effective behavioral treatments for ABLD have yet to be documented, a decision was made to study the effects of LSVT LOUD with a woman with ABLD.

## Objective

The purpose of this study was to document the effectiveness of an intensive behavioral voice treatment, LSVT LOUD (Alharbi et al., 2019; Bryans et al., 2021; Sapir et al., 2002) administered to a speaker with ABLD who had received an injection of BoNT in each vocal fold 2 to 3 months prior to treatment. This case study will document the immediate and long-term effects of a behavioral treatment on vocal quality and loudness for a woman with ABLD.

## Case Study Participant



The participant (M.Z.) was a 47-year-old registered nurse employed in a hospital setting.

Initial onset of ABLD symptoms was noted in 2017 and described as breathy vocal quality, occurring at the end of her work shift, following a typical day of voice use. Over time, when her voice interfered with her job effectiveness, she sought medical attention. M.Z. had an initial visit with an otolaryngologist (ENT) in March 2018 with a complaint of vocal hoarseness. She was evaluated using fiberoptic laryngoscopy. Findings indicated moderate edema and erythema in the pyriform sinuses, interarytenoid region, and aryepiglottic folds. The patient was treated for gastroesophageal reflux disease with esophagitis. The ENT prescribed omeprazole, Nasacort AQ, and a low-acid diet. This resulted in no improvement in vocal function.

M.Z. returned to the ENT in August 2019 with worsening symptoms. Reflux precautions were reiterated and she was prescribed Pepcid at bedtime. M.Z. followed up with a colleague of this ENT practice a week later, as the report stated that her “symptoms were constant, moderate, and unchanged.” The patient was referred for allergy evaluation. M.Z. was diagnosed with allergic rhinitis due to pollen with positive skin test results for tree, grass, ragweed, weed pollen, mold, cat, dog, and dust mites. She was prescribed Nasacort and ipratropium bromide via nasal spray.

Following worsening voice quality, M.Z. was subsequently evaluated in September 2019 by a voice-specialist ENT who noted her “breathy breaks” in connected speech. This specialist performed videostroboscopy and diagnosed her with ABLD 2 years after her symptoms were first noted. The ENT recommended botulinum toxin injections. He provided education on the typical recurrent injection cycle and the usual need to titrate the injection cycle. Possible adverse reactions were discussed, including the expected period of breathiness and mild to moderate dysphagia. He also recommended voice therapy of one to two sessions per week × two to six sessions and referred her to a speech-language pathologist to “optimize voicing efficiencies.” The patient pursued this avenue and was seen for one session. The speech-language pathologist provided support resources, education on SD, and vocal hygiene tips. She did not recommend treatment, claiming that there was no evidence-based behavioral voice therapy treatment for ABLD.

M.Z. reported that over time her voice became increasingly dysphonic. Her voice affected her ability to perform work duties such as phoning doctors for orders or communicating with staff and patients. Wearing a facemask further reduced her intelligibility to the point where she had to resign from her position.

*My voice declined to the point that air was just escaping whenever I tried to talk. My speech was exhausting, unintelligible, broken and breathy. I felt functionally mute.*

At her home, her family had a hard time hearing her; she had to use strategies, such as hand clapping, to get their attention. M.Z. came to realize that vocal fold injections would be her only option for treatment, but the COVID-19 pandemic suspended most ambulatory medical procedures during this time. As office treatments returned, M.Z. moved forward to schedule the first set of BoNT injections. M.Z. was examined by the ENT with fiberoptic laryngeal stroboscopy who reported “her voice is very breathy on connected speech segments” noting “large abductor spasms on connected speech; full range of motion; able to close the glottis on coughing; her left vocal fold abductors with more activity than right.” The ENT treated her voice with BoNT Type A injection of the left PCA muscle in March 2021 ( Supplementary Material 1: Voice Sample 3.22.21. Botox Injection Day ). A second injection was administered to the right PCA muscle 3 weeks later.

M.Z. reported no improvement in her voice following the initial BoNT treatment. Before receiving a second round of BoNT injections, the ENT referred the patient to a movement disorder neurologist. The neurologist informed M.Z. that with no other neurological deficits, no medication was available for her vocal dystonia. However, the neurologist recommended that she seek LSVT LOUD therapy, indicating that she had found it to be effective for some of her patients with ABLD.

## Method

M.Z. was made aware that there was no published evidence that LSVT LOUD therapy would be effective for her voice disorder and that this application could be applied in a clinical investigation study. Consent was obtained from the participant. M.Z. was accepted for evaluation and a trial of intensive voice therapy was initiated. With the approval of Long Island University's Institutional Review Board (IRB Protocol ID: 21/09-113), this client became a clinical case study. The aim of this study was to assess the effects of intensive behavioral voice therapy following an initial dose of bilateral injection of BoNT. Improvements in vocal quality and reductions in voice breaks were evaluated over the 4-week period of intensive therapy and at intervals of 1, 3, 6, and 12 months posttherapy. This case study encompasses a baseline level of function, followed by a treatment phase, followed by a maintenance phase.

## Study Setting

M.Z. was evaluated virtually within the university clinic program at Long Island University, Post Campus (LIU Post). The participant had been diagnosed with ABLD prior to her being evaluated by a speech-language pathologist at the university. The diagnosis of ABLD is not straightforward and medical diagnosis is often delayed, as it was in this case. Simonyan et al. (2021) recommended that the findings of a multidisciplinary clinical assessment consisting of a detailed case history, auditory-perceptual testing, nasoendoscopy, and neurologic examination be considered as part of a differential diagnostic process. M.Z. was evaluated by two otolaryngologists, a neurologist, and a speech-language pathologist during the diagnostic process. The diagnosis of ABLD was rendered by an ENT 2 years post-onset of voice symptoms. Based on the previous medical findings and our own case history and perceptual evaluation, this diagnosis was verified.

Therapy was executed according to procedure for all clients at the Ladge Speech and Hearing Center at LIU Post. Due to COVID-19 restrictions in 2021, treatment was administered virtually utilizing the Zoom platform. LSVT LOUD/teleLOUD has been found to be equally efficacious when delivered in person and via telepractice as long as the protocol is maintained (Constantinescu et al., 2011; Halpern et al., 2012; Howell, Tripoliti, & Pring, 2009; Theodoros, Hill, & Russell, 2016; Theodoros & Ramig, 2011; Tindall et al., 2008). Telepractice had been well established in the literature given the need for virtual access for voice therapy for people with PD. As clinical voice care via telepractice became essential during the COVID-19 pandemic, the reliability of auditory-perceptual voice treatment within such platform was corroborated (Dahl et al., 2021). LSVT LOUD/teleLOUD was administered by three LSVT LOUD–certified graduate clinicians and supervised by a LSVT LOUD/teleLOUD speech-language pathologist/clinical educator.

## Instrumentation

The participant and clinicians used laptop computers in their homes within a quiet, private space. Computer microphones and speakers without earphones were used. The volume of all devices was set to the loudest setting. The laptops were positioned on a desk in front of the participant and clinicians so that their faces filled the Zoom screen. The clinicians used a sound level meter to judge relative levels of amplitude from session to session. Instrumentation for data collection included a stopwatch, BAFX3370 digital sound level meter, and the iOS Tuner application by Piascore, an app endorsed for reliability and consistency in the LSVT LOUD training course. For the purpose of analysis of pitch range, frequency measures were converted to semitones.

## Intervention

The clinical voice evaluation was conducted 2 months after her second BoNT injection. At the time of the evaluation, M.Z. described her voice as “extremely breathy and weak.” Based on clinical judgment, she presented with a moderate-to-severe voice disorder secondary to ABLD. M.Z.'s voice was characterized by breathiness, straining to produce voice, and notable difficulty transitioning from voiceless to voiced phonemes. There were aphonic periods with frequent abductor voice breaks; however, she exhibited intermittent phonation on fillers (i.e., um, yeah) and with laughter. Initially, M.Z. was unable to vocalize in a conversational manner over teletherapy. The Zoom platform could not pick up her essentially aphonic voice and conversational intelligibility was markedly reduced. Therefore, case history was obtained via forms, medical records, and written format.

### Baselines

As vocalizations for vowel phonation were audible, baseline measures and stimulability testing were completed per protocol for the LSVT LOUD intensive program. Included for reference are the charted baseline data from the daily voice exercises on day 1 of therapy ( Table 1 ).

**Table 1:** Baseline data on day 1 of intensive therapy

Baselines	Duration: average (range)	Frequency: average (range)
Sustained vowel /a/	12 s (4–17 s)	80 dB SPL a (76–85 dB SPL)
Maximum high frequency within a pitch glide		553 Hz
Maximum low frequency within a pitch glide		213 Hz
Range in semitones		17
Functional phrases		76 dB SPL (69–83 dB SPL)
Reading words		80 dB SPL
Conversation		72 dB SPL

a Decibel sound pressure level.

Voice quality was perceptually measured pretreatment using the GRBASI scale by a speech-language pathologist unassociated with the study. The GRBASI scale is a perceptual-acoustic measure to assess overall grade, roughness, breathiness, asthenia, strain, and instability in all types of voice disorders regardless of etiology. The sub-scale rating is a 4-point scale: 0 (normal), 1 (slight), 2 (moderate), and 3 (severe) for each measured component (Yamauchi et al., 2010). Pretreatment findings for the study participant were as follows: grade 2, roughness 0, breathiness 2, asthenia 1, strain 2, and instability 2.

The Voice Handicap Index (VHI) (Jacobson et al., 1997) was utilized as a subjective patient-reported outcome measure at baseline. The VHI is a validated, self-administered 30-item questionnaire that considers the impact of voice disorders on a subject's daily life (Jacobson et al., 1997). This outcome measure is commonly used for a variety of voice disorders. The VHI has five response levels ( *0—never; 1—almost never; 2—sometimes; 3—almost always; 4—always* ). M.Z. indicated *4—always* for the majority of the items presented to describe the functional (i.e., *My voice makes it difficult for people to hear me* ), physical (i.e., *I run out of air when I talk* ), and emotional restrictions (i.e., *I am tense when talking to others because of my voice* ) of her voice. This resulted in a total score of 103 out of a possible 120, placing self-perception of her voice handicap in the severe category.

### Treatment Phase









In exercise 1, M.Z. sustained vowels as long as possible for 15 trials, following the clinician's model. Pitch instability was present during sustained phonation, yet voicing was continuous at an intensity level of 80 dB measured by the clinicians using a BAFX3370 digital sound level meter. Clinicians provided hand gestures to maintain high effort levels. Her achieved intensity level in dB sound pressure level (SPL) and duration in seconds were shared to motivate subsequent trials.

Exercise 2 involved M.Z. performing pitch glides from modal pitch to highest possible pitch, and from modal pitch to lowest possible pitch. Each glide was sustained for 5 seconds with 15 repetitions following the clinician's model. Data were recorded in Hz, as obtained in the iOS Tuner application, and shared with the participant for motivation in subsequent trials.

Exercise 3 consisted of repetition of functional phrases at increased loudness levels ( Table 2 ). M.Z. was asked to read the list of 10 phrases five times each with the prompt to “Think Loud.” Volume for functional phrases was recorded in dB SPL with a sound level meter. Vocal abductor voice breaks were evident in this speaking exercise, when transitioning from voiceless to voiced consonants. In order to maximize M.Z.'s success, the number of voiceless consonants was reduced by using alternate vocabulary, i.e., replacing “ *How are you feeling* ?” with “ *Are you doing well* ?” These 10 phrases were used throughout the 4-week treatment period.

**Table 2:** Daily functional phrases are client-specific phrases formulated for daily voice exercises

Daily functional phrases
1 Where are you going? 2 Are you doing well? 3 Dinner is ready! 4 You are welcome! 5 I'd like you to clean up! 6 I'm so proud of you! 7 Nice to meet you! 8 Will I see you later? 9 93, fill it up! 10 Answer the phone, please!

Exercise 4 involved reading single word lists of salient content for 25 minutes. Word lists for M.Z. included topics on health, medicine, gardening, and cooking. Approximately 200 words were read aloud with high effort. Words that began with voiceless consonants were avoided as much as possible to reduce the opportunity for voice breaks during vocal fold abduction.

M.Z. was encouraged to hydrate throughout all vocal exercises.

Treatment followed the LSVT LOUD intensive therapy protocol for 16 sessions over 4 consecutive weeks. These 1-hour sessions targeted increasing volume, expanding vocal range, and calibrating awareness in speaking with high effort to conversational speech. Each therapy session consisted of two parts: the core LOUD exercises for 30 minutes and the hierarchical exercises for 30 minutes. The LOUD core exercises involved sustaining the isolated vowel /a/, high- and low-pitch glides, and functional phrases. To facilitate generalization of a loud voice, the hierarchical speech exercises consisted of oral readings and structured speech activities of increasing length and complexity over the 4-week period. LSVT LOUD intensive therapy was administered with additional provisions owing to the diagnosis of ABLD (see Text Box 1 ).

**Text Box 1:** LSVT LOUD therapy modifications for ABLD

● The oral reading lists for the hierarchical exercises concentrated on words, phrases, and sentences with initial vowels and voiced consonants ● Isometric exercises were incorporated to utilize hyperfunction for vocal fold adduction (Angadi, Croake, & Stemple, 2019) by exerting internal glottal pressure to reduce the occurrence of abductor spasm ● Vocal fold approximation was initiated by vocalizing /a/ to generate voice onset from sustained phonation through the articulation of the utterance, i.e., /a/muvi/ ● The duration of voiceless consonants was shortened to quickly reach the vowel in an attempt to decrease the opportunity for abductor spasm

Week 1 consisted of increasing average speech amplitude and amplitude range, extending maximum phonation time (MPT), and producing vocal glides to extend pitch range. Verbal tasks consisted of word- and phrase-level oral readings. Spontaneous speech was perceptually breathy ( Supplementary Material 2: Voice Sample , Spontaneous Speech 6.23.21. Day 4 of LSVT). The participant was producing customized functional phrases with effort as prompted by the clinician ( Supplementary Material 3: Voice Sample , Functional Phrases 6.24.21. Day 4 of LSVT). As M.Z. was motivated and able to phonate and increase vocal loudness without complaints of vocal pain or strain by the end of the first week, therapy continued.

Week 2 consisted of continuation of vocal exercises to focus on improving vocal volume and endurance, strengthening her voice, and reducing the occurrence and intensity of voice breaks within sentence-level productions. During this treatment period, less clinician cueing was required to maintain high effort, and M.Z. reported that the exercises were feeling easier to accomplish. M.Z. was challenged with carryover speaking assignments, which she did conscientiously. Functionally, M.Z. reported having a successful telephone conversation with a family member during the week ( Supplementary Material 4: Voice Sample 7.6.21. Day 9 of LSVT ).

In week 3, exercise performance required no clinician verbal cueing for effort, but M.Z. was provided with motivational feedback on her outcomes for each trial of the core exercises. She was encouraged to project her voice but refrain from shouting, as evidenced in her functional phrases ( Supplementary Material 5: Voice Sample , Functional Phrases 7.7.21. Day 10 of LSVT). For the first time, M.Z. was able to extend MPT from 12 to 20 seconds at 87 dB SPL. The vocal range of the participant continued to expand. The third week of therapy focused on paragraph level within hierarchical speech tasks. M.Z. preferred speaking extemporaneously over a reading task as this felt more natural. The tasks addressed lengthy verbalization, such as describing picture scenes, telling jokes, defining proverbs, and completing stories. Functionally, M.Z. reported that she spoke audibly with her daughter's physician and was able to converse with a neighbor. She stated that she was gaining confidence in using her voice.

In week 4 it was noted that M.Z. used her voice with good functionality. Therapy tasks at the conversational level included explaining viewpoints, telling short stories, and describing life events. M.Z. was able to produce conversational speech at a consistent amplitude of 85 dB SPL. Functionally, M.Z. was able to answer the phone, engage in open conversation, and call her family members in other rooms of her home.

## Results

Data were recorded for each parameter over 16 teletherapy sessions in 4 weeks. Therapy was conducted from June to July 2021, with follow-up sessions via teletherapy at 1, 3, 6, and 12 months posttreatment to determine maintenance, regression, or improvement in voice function over time ( Table 3 ).

**Table 3:** Data summary for duration and amplitude of sustained vowels across 16 sessions and 4 follow-up sessions

Treatment day	Sustained vowel duration average in seconds	Sustained vowel duration range in seconds	Sustained vowel amplitude average in dB SPL	Sustained vowel amplitude range in dB SPL	Level of cue provided	Perceived effort by participant
1	12	4–17	80	76–85	Mod	Mod
2	15	13–18	85	81–90	Min	Mod
3	14	11–17	85	81–90	None	Mod
4	15	11–19	83	82–86	None	Mod
5	14	11–17	85	80–89	None	Mod
6	14	12–16	84	80–87	None	Easy–Mod
7	17	14–19	86	80–91	Min	Easy
8	19	11–22	85	82–88	None	Easy
9	21	20–23	86	82–90	None	Easy
10	21	19–24	88	85–91	None	Easy
11	21	19–23	87	85–90	None	Easy
12	20	19–23	87	84–90	None	Easy
13	18	17–19	88	86–90	None	Easy–Mod
14	21	19–22	88	86–90	None	Easy–Mod
15	19	18–21	87	84–90	None	Easy–Mod
16	20	19–22	87	84–90	None	Easy
1 mo post	24	23–26	88	85–91	None	Easy
3 mo post	24	23–28	81	78–84	None	Easy
6 mo post	25	23–28	84	83–85	None	Easy
12 mo post	30	26–32	83	81–87	None	Easy

### Maximum Phonation Time

Over the 4-week treatment period, sustained vowel duration gradually increased from 12 to 20 seconds over the first 2 weeks of treatment. MPT was maintained at this level for the remainder of the therapy sessions. With reported daily exercise, the client was able to sustain the /a/ vowel for 24 seconds at 1 month posttherapy. Sustained vowel duration was maintained at 3 to 6 months post, with M.Z. admitting to doing the voice exercises “only occasionally.” Sustained vowel duration continued to improve posttherapy with no execution of maintenance voice exercise ( Table 3 ).

### Frequency Range

M.Z.'s frequency range expanded significantly during vocal glides. At baseline, her frequency range from high to low was 16.5 semitones as measured using the iOS Tuner application. At the end of the treatment period, her frequency range had expanded to 30 semitones ( Table 4 ).

**Table 4:** Average high- and low-frequency and average semitone range for all therapy sessions

Treatment day	1	2	3	4	5	6	7	8	9	10	11	12	13	14	15	16
Average high frequency achieved in hertz	553	625	650	601	585	593	640	787	783	820	808	790	819	817	820	830
Average low frequency achieved in hertz	213	207	191	187	200	180	180	183	173	172	159	171	161	151	144	147
Average semitone range	16.5	19	21	20	18	21	21	22	26	27	28	26	28	29	30	30

Source: Diaz G. Semitone Calculator. Available at: https://www.omnicalculator.com/other/semitone . Accessed November 16, 2023.

### Vocal Quality

Perceptually, the occurrence and severity of vocal spasms gradually decreased over the course of treatment. At the start of therapy, inappropriate vocal spasms and instability were evident during all vocal tasks and her voice was essentially aphonic in conversational speech. The speech-language pathologist re-assessed voice quality using the GRBASI posttreatment. Voice quality improved across subcategories ( Table 5 ).

**Table 5:** GRBASI 4-point rating scale

	Component	Description	Pretreatment	Posttreatment
G	Grade	Degree of hoarseness of the voice	2	1
R	Roughness	Impression of irregularity of the vibration of the vocal folds	0	0
B	Breathiness	Degree to which air escaping from between the vocal folds can be heard by the examiner	2	1
A	Asthenia	Degree of weakness heard in the voice	1	0
S	Strain	Extent to which strain or hyperfunctional use of phonation is heard	2	0
I	Instability	Changes in voice quality over time	2	1

Note: Rating scale: 0, normal; 1, slight; 2, moderate; 3, severe.

Source: Yamauchi et al (2010).

M.Z. completed the VHI three times during the course of the study. Baseline scores reduced from the severe range to normal limits ( Table 6 ). As noted in the patient-reported outcome measure, M.Z.'s perception of voice functionality improved. M.Z. used her strong voice regularly throughout the day and reported no residual intelligibility issues, even over the telephone. Her independence and quality of life improved, as she was able to be heard while wearing a face covering or in an environment with background noise. Upon therapy completion, M.Z. was encouraged to continue the daily exercises at a maintenance level of six repetitions of isolated vowels and pitch glides, to read her functional phrases, and to read aloud with a strong voice for 10 to 15 minutes every day. An appointment was scheduled for a 1-month follow-up.

**Table 6:** Results of the Voice Handicap Index (VHI) at baseline, immediately posttherapy, and at 3 mo posttherapy

VHI	Baseline pre-Tx 06/21/2021	Immediately post-Tx 07/15/2021	3 mo post-Tx 10/19/2021
Functional restrictions	39	16	1
Physical restrictions	28	11	0
Emotional restrictions	36	11	0
Overall score and severity rating	103 = severe	38 = moderate	1 = WNL

Abbreviations: Tx, therapy/treatment; WNL, within normal limits.

Source: Jacobson et al., 1997.

## Maintenance Phase

### One Month Posttherapy

Objective data for sustained vowel /a/ increased from 20 to 24 seconds. High-frequency glides increased from 830 to 850 Hz (0.41 semitones). Conversational speech averaged 84 dB and minimal spasms were noted perceptually.

### Three Months Posttherapy

M.Z. presented with a voice free of breathiness and pitch instability. Sustained phonation remained steady at 24 seconds. Again, high frequency increased from 850 to 905 Hz (1.1 semitones). Conversational speech averaged 84 dB SPL. Vocal breaks were nearly undetectable. She admitted that she was performing maintenance voice exercises infrequently but was using her voice fully and functionally. At this time, M.Z. was given the VHI to reevaluate how her voice restricts her life. She scored *0—never* for all situations, but one measure of *1—almost never* with a total score of 1, indicating her perception that her voice does not restrict her function ( Table 6 ).

### Six Months Posttherapy

Auditory perceptual analysis revealed speaking quality to be within normal limits in conversational speech. M.Z. sustained phonation at 25 seconds. High frequency rose from 905 to 940 Hz (0.66 semitones). Conversational speech averaged 80 dB with no vocal prompts and without any regular voice exercise other than typical daily talking.

### Twelve Months Posttherapy



M.Z. maintained a vocal quality as described at 6 months posttherapy. Sustained phonation averaged 30 seconds. High frequency reduced from 940 to 907 Hz (−0.62 semitones). Vocal volume in connected speech was at 83 dB, which is well within normal limits. Vocal volume in connected speech appeared to be effortless. Conversational discourse revealed no perceptual evidence of LD. M.Z. did not engage in maintenance vocal exercises. She had not received any BoNT injections within the last 15 months ( Supplementary Material 6: Voice Sample , Functional Phrases 6.21.22. One Year Post-LSVT).

## Discussion

As previously discussed in the introduction, the efficacy of LSVT LOUD has been widely researched and documented within the population of individuals with PD as well as in patients with various motor speech disorders and diagnoses (Baldanzi et al., 2022; Lowit et al., 2020; Lu et al., 2013; Mahler & Jones, 2012; Mahler & Ramig, 2012; Wenke et al., 2008). However, to date, it has not been studied in cases of abductor LD.

ABLD is a focal dystonia that affects the vocal folds and results in dysphonia. There is strong evidence that vocal function exercises are efficacious for a variety of voice disorders to strengthen or remediate voice production (Angadi, Croake, & Stemple, 2019). This may be attributed to the way vocal function exercises involve the physiologic interplay between the respiratory source, phonatory musculature, and resonance chambers of the vocal tract. Thomas and Stemple (2007) noted that physiologic approaches were superior to other voice treatments. The symptoms of ABLD, however, have not proven to be alleviated by physiologic approaches (Simonyan et al., 2021). We hypothesized that the integrated effects of physiologic, symptomatic, and behavioral approaches combined with intensity and effort might result in a more efficacious result than one method or another.

In this case study, we explored the treatment effects of intensive voice therapy applied to a speaker with ABLD following initial injections of BoNT. The injections had not yielded any perceptual improvement in voice quality or in reduced vocal symptoms, which is not uncommon following first injections (Blitzer et al., 1998; Simonyan et al., 2021). Objective measurements obtained during intensive behavioral therapy using the LSVT LOUD protocol revealed a gradual, steady increase in M.Z.'s vocal intensity, vowel duration, frequency range, and voice quality over the 4-week treatment period with noticeable reduction in perceptually detectable aphonic breaks. In addition, posttreatment outcome measures demonstrated maintenance of these gains with normal daily voice use for 12 months posttreatment.

The vocal symptoms presented with abductor LD necessitated some modification of the LSVT LOUD protocol. M.Z.'s difficulty transitioning from voiceless to voiced sounds within words and phrases led the first author to reduce the occurrence of vocal abduction within therapy tasks by reducing the number of these transitions in practice materials. Reading lists were revised where possible to minimize the inclusion of voiceless phonemes. Words and phrases that began with voiceless consonants were omitted from the daily functional phrases and reading exercises. Additionally, M.Z. was coached to minimize duration of voiceless sounds and begin voicing loudly on the next vowel. This instruction proved to be successful as a method to reduce the occurrence of vocal interruptions which began to gradually decrease in number and severity in weeks 3 and 4 of therapy. By facilitating more continuous phonation during practice exercises, M.Z. was able to maintain improved vocal quality within “off the cuff” utterances during the therapy session.

A pertinent component for improved vocal function with therapy involved executing daily voice exercises and repetitive functional phrases at home. The participant was diligent in completing home exercises once daily on therapy days, and twice daily on nontherapy days as directed for 10 to 15 minutes. The home practice consisted of sustaining the vowel /a/, low- and high-pitch glides, and reading of functional phrases. Vocal hygiene focused on frequent hydration. The participant was instructed to refrain from whispering, as she admitted it was a common practice prior to treatment, erroneously assuming that whispering would be less damaging to her voice.

M.Z. was very satisfied with her vocal quality and minimal presence of vocal abductions in conversational speech at the end of the treatment period. Her independence and quality of life improved. She was using her strong voice throughout the day and reported no residual intelligibility issues, even over the telephone. She was heard while wearing a face covering and in environments with background noise. These gains were maintained over the next 12 months with no structured vocal practice aside from speaking. She stated, “It's like this never happened. My voice feels 99.9%.”

M.Z. self-selected this treatment at her neurologist's suggestion. No one anticipated this excellent outcome. The degree of improvement in vocal function in the subject is remarkable and exceeds the degree of improvement reported in previous studies (Baumgartner et al., 2001; Levy et al., 2020; Sapir et al., 2002). Not only were vocal characteristics of ABLD reduced, but the results were maintained for many months posttreatment.

Much still needs to be discovered regarding the causes of ABLD, its physiological underpinnings, and development of relevant treatments. Dysfunction of the basal ganglia networks and the inferior parietal cortex are associated with LD which may adversely affect brain plasticity (Simonyan et al., 2021). A knowledge gap exists regarding the role of primary versus compensatory neural abnormalities in pathophysiology (Simonyan, 2017; Simonyan et al., 2021).

Fox et al. (2006) reported that focusing on strengthening respiratory and phonatory systems positively impacts a range of neural networks associated with neurological disorders. Administration of a behavioral protocol based on the principles of brain plasticity may be responsible for the remarkable results seen in this study. The principles of neuroplasticity, namely, frequency, force, repetition, effort, accuracy, and saliency, contribute to lasting improvements in changes in brain functions (Kleim & Jones, 2008; Ludlow et al., 2008).

M.Z. demonstrated vocal improvements within the first week of treatment which motivated her to continue. The materials used in treatment reflected her specific interests contributing to saliency. M.Z. followed the clinicians' model utterances precisely with force and effort over many repetitions. We did not have the means to measure changes in brain function associated with ABLD, but the perceptual effects were better than expected and longer lasting. Unlike progressive disorders such as PD and multiple sclerosis, regular structured practice appeared not to be needed to maintain normal speech quality.

It is possible that BoNT injections prior to the initiation of LSVT LOUD may have contributed to her positive response to the treatment. Continued maintenance of the gains, however, extended well past the effects of BoNT for ABLD, which is typically 3 months.

This study draws attention to the potential benefit of intensive, effortful behavioral voice therapies like LSVT LOUD. Further research is needed to corroborate these findings.

## Study Limitations

As a case study, the results obtained here should be viewed as preliminary in the hope that this approach will be replicated by others. While there is clear perceptual and behavioral evidence of lasting improvement in vocal loudness, vocal quality, and speech intelligibility for M.Z., the acoustic data collected may not be reliable as mouth-to-microphone distance was not verified by the participant or the clinicians, as treatment was administered via teletherapy. In addition, frequency data were measured by an iOS application and its calibration was not verifiable. Variables would likely be more reliably controlled if this study could be replicated with multiple subjects, in-person and with the use of the LSVT Coach System. We did not have the means to document physiological brain changes to brain function that might have occurred. This would be an interesting addition for future research.

## Conclusions

The results from this case study demonstrate that LSVT LOUD was an effective clinical intervention for this individual with ABLD following initial BoNT injection. Continued research using a larger pool of subjects in a randomized controlled study is suggested to further support the efficacy of applying intensive voice therapy to speakers with ABLD. If so, the need for invasive vocal fold injections that have short-term benefits could be avoided. An additional query is whether a speaker with ABLD would benefit from other intensive behavioral treatments.
