# Supplementary material for: Intensive Voice Treatment following Botulinum Neurotoxin Injection for a Speaker with Abductor Laryngeal Dystonia: An Exploratory Case Study
Source: Semin Speech Lang. 2024 Feb 28;45(2):137–51. doi: 10.1055/s-0044-1779509 (PMC10957285; doi:10.1055/s-0044-1779509)
Supplement: Supplementary file 7 — Supplementary Material [file 10-1055-s-0044-1779509-s230029clina.pdf]

Supplementary Material 1: Voice Sample 3.22.21 which demonstrates M.Z.'s vocal quality after the initial BoNT injection of the left PCA. This sample preceded intensive voice therapy.

Supplementary Material 2: Voice Sample 6.23.21 which demonstrates M.Z.'s vocal quality during week 1 of intensive therapy.

Supplementary Material 3: Voice Sample 6.24.21 of M.Z. reading her daily functional phrases at the end of week 1 of intensive therapy.

Supplementary Material 4: Voice Sample 7.6.21 of M.Z. at the start of week 3 of intensive therapy.

Supplementary Material 5: Voice Sample 7.7.21 of M.Z. reading her daily functional phrases in week 3 of intensive therapy.

Supplementary Material 6: Voice Sample 6.21.22 of M.Z. reading her daily functional phrases 1 year after completing intensive voice therapy. M.Z. did not receive any additional BoNT treatments over the course of the year, nor was she performing vocal exercises.
